# Aspirin's Role in Reducing Oxidative Stress: Implications for Cognition and Heart Function in Autism Spectrum Disorder

**DOI:** 10.1155/vmi/4089667

**Published:** 2025-08-28

**Authors:** Mostafa Mana, Zohreh Ghotbeddin, Kaveh Khazaeel, Abdolvahed Moarabi, Hoda Parsa

**Affiliations:** ^1^Department of Basic Sciences, Faculty of Veterinary Medicine, Shahid Chamran University of Ahvaz, Ahvaz, Iran; ^2^Stem Cell and Transgenic Technology Research Center, Shahid Chamran University of Ahvaz, Ahvaz, Iran; ^3^Department of Clinical Sciences, Faculty of Veterinary Medicine, Shahid Chamran University of Ahvaz, Ahvaz, Iran; ^4^Department of Biology, Faculty of Sciences, Shahid Chamran University of Ahvaz, Ahvaz, Iran

**Keywords:** aspirin, autism, cognition, left ventricle hypertrophy, rat, social behavior, VPA

## Abstract

**Background:** Autism spectrum disorder (ASD) is characterized by impairments in social communication and the presence of additional conditions such as heart disease. Oxidative stress has been linked to the severity of autism, suggesting a potential role for antioxidants in mitigating its effects. Aspirin, an antioxidant and anti-inflammatory drug, has shown protective effects on heart function. This study aimed to investigate the effects of aspirin on cognition, social behavior, and left ventricular hypertrophy in adult male rats following induction of an autism model with valproic acid (VPA).

**Methods:** Pregnant Wistar rats were divided into four groups: control, VPA, aspirin, and VPA + aspirin. VPA was administered on the 12th day of pregnancy to induce the autism model. Offspring in the aspirin group received aspirin after weaning. Social behavior and cognition were assessed in adulthood, and left ventricular thickness and heart function were evaluated using echocardiography. Oxidative stress markers in the hippocampus were also measured.

**Results:** The results showed that VPA-exposed rats exhibited decreased social behavior and cognition compared to the control group. However, aspirin treatment improved social interaction and cognition in the VPA-exposed rats. Left ventricular thickness, heart rate, and volume increased in the VPA group, while aspirin treatment mitigated these changes. Additionally, VPA exposure led to increased oxidative stress, which was reduced by aspirin treatment (all cases *p* < 0.05).

**Conclusion:** VPA-induced autism model during pregnancy resulted in disturbances in social behavior, cognition, and heart function in offspring, accompanied by increased oxidative stress. Aspirin treatment showed improvements in social behavior, cognition, and cardiac parameters, possibly by reducing oxidative stress markers. These findings suggest a potential therapeutic role for aspirin in ameliorating the behavioral and cardiac issues associated with VPA-induced autism model.

## 1. Introduction

Autism spectrum disorder (ASD) consists of a range of neurological and developmental conditions [1]. Individuals with ASD frequently experience challenges in social communication and interaction, along with demonstrating restricted or repetitive behaviors or interests [1]. Despite extensive research in this area, the exact cause of ASD remains unknown, and there have been limited advances in treatment. People with ASD may experience various learning, movement, or attention disorders [2]. In addition to behavioral changes such as hyperactivity, social interaction impairments, reduced exploratory activity, and increased repetitive behaviors reported in these people, comorbidities such as heart disease are also common with autism [3].

According to studies, the probability of diabetes, high cholesterol, and heart disease is higher in ASD compared to the normal people [4]. While the link between autism and the risk of obesity and gastrointestinal diseases has been well established, there is limited information regarding the impact of autism on metabolic heart health, which requires further research and study. A meta-analysis study shows that people with autism are more susceptible to the developing metabolic heart diseases, including diabetes, dyslipidemia, and cardiovascular disease (CVD) [5]. Cheng and colleagues reported in 2020 that reduced heart rate variability indexed by low heart rate is a potential biological indicator for ASD [6]. However, the findings are contradictory, as decreased or unchanged parasympathetic activity and heart rate have been reported in children with autism [6]. In patients with ASD, reduced heart rate variability may also intersect with cardiovascular issues, as cardiac hypertrophy is frequently observed in patients with heart failure (HF), highlighting potential shared physiological mechanisms [7].

Although autism affects humans, animal models of this disorder can help discover the cause of autism and test therapeutic factors [8]. One of the common animal models for inducing autism is exposure to antiseizure drugs such as valproic acid (VPA) [9].

Oxidative stress is generated because of the high generation of reactive oxygen species (ROS) and reactive nitrogen species (RNS), two primary indicators of redox [10]. ROS/RNS play a role in the pathogenesis of cardiac hypertrophy [11]. Mei Li et al. in 2023 demonstrated a significant correlation between left ventricular mass index and ROS/RNS levels in 104 patients with hypertension and left ventricular hypertrophy [12].

Increased oxidative stress is one of the main mechanisms of behavioral impairment in autism [13]. It has been reported that autistic patients and animal models of an autism exhibit high lipid peroxidation and reduced expression of detoxification factors (such as glutathione) and antioxidants involved in the defense system against ROS [14]. Furthermore, a positive correlation has been observed between decreased levels of antioxidants or increased ROS and the severity of autism [15].

Considering the increased oxidative stress and lipid peroxidation in autism, utilizing antioxidants like aspirin may help alleviate symptoms and mitigate the complications of autism. Concerning the antioxidant properties of aspirin, research by Durak et al. (2001) showed that both high and moderate doses of aspirin markedly elevate toxicity in the myocardium of Indian piglets, whereas a low dose (10 mg/kg) exhibits protective benefits [16]. Chen et al. also investigated the effects of aspirin on acute lung injury induced by hypoxia and found that aspirin has anti-inflammatory and antioxidant effects by inhibiting the NF-kB signaling pathway [17]. Aspirin is a nonsteroidal anti-inflammatory drug that inhibits the inflammatory response and lowers the levels of inflammatory markers like C-reactive protein, tumor necrosis factor-alpha, and IL-6 [18]. It may also lessen oxidative stress and safeguard against oxidative damage [19]. Certain preclinical and clinical findings indicate the positive effects of aspirin in behavioral disorders [20].

Aspirin was chosen for this research because of its dual pharmacological effects as an antioxidant and anti-inflammatory agent, which corresponds with the multifactorial pathology of ASD that includes oxidative stress, neuroinflammation, and cardiac dysfunction. Its recognized cardioprotective benefits—including reduction of left ventricular hypertrophy and improvement of vascular endothelial function—further underscore its significance with ASD-related cardiovascular issues [21]. Moreover, aspirin has been found to influence mitochondrial function and glial activity, which are increasingly associated with neurodevelopmental disorders [22]. Its positive safety profile, cost-effectiveness, and practical applicability make it a realistic option for preclinical repurposing initiatives in ASD models.

Considering that one of the comorbidities associated with autism, in addition to behavioral and social problems, is CVD, there is limited information about the effect of autism on cardiac function, and the findings are contradictory. On the other hand, many earlier studies mention aspirin's protective role in cardiac function, although findings in this area are also inconsistent and vary with dosage. Consequently, this research aimed to examine the effect of aspirin after the creation of an autism model using VPA on cognitive function, social interactions, and left ventricular hypertrophy in adult male rats.

## 2. Methods

### 2.1. Animals and Grouping

The Institutional Ethics Committee at Shahid Chamran University of Ahvaz granted approval for the present study (EE/1400.2.24.42815/Scu.ac.ir) in compliance with the ARRIVE guidelines. All processes were conducted in accordance with the relevant protocols and regulations. To reduce stress, pregnant rats were brought to the laboratory a week before Parturition and their autistic offspring were born, cared for daily, and experiments were conducted with minimal noise and vibration. Male and female rats were kept in a ratio of 3:1 for mating in standard laboratory conditions, humidity 50% and temperature 23 ± 2 Celsius with a 12-h light and dark cycle and were housed in the separate cages. All the animals had free access to food and water.

**Figure 1:**
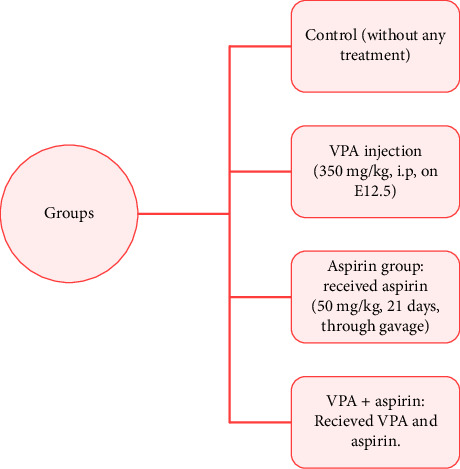
Schematic diagram of the experimental groups.

The female rats being tested were grouped together for 1 week to align their sexual cycles. The vaginal smear technique was employed to assess the pregnancy status of the rats. Our standard for identifying the half-embryonic day (E0.5) was the detection of spermatozoa in the sample. After this criterion was observed, the female rat under study was considered as a definite positive and separated from the male rat. Pregnant rats were housed in separate cages and were randomly divided into four groups as follows:

1. VPA + Saline Group: Pregnant rats were injected intraperitoneally with VPA at a dose of 350 mg/kg (3.3 mL/kg) on embryonic day 12.5 (E12.5). The offspring received saline through gavage after weaning for a duration of 21 consecutive days.
2. VPA + Aspirin Group: Pregnant rats were injected intraperitoneally with VPA at a dose of 350 mg/kg (3.3 mL/kg) on E12.5. The offspring received aspirin at a dose of 50 mg/kg through gavage for 21 consecutive days after weaning.
3. Vehicle + Saline Group: Pregnant rats were injected with the vehicle (saline) on E12.5, and the offspring received saline through gavage after weaning for a duration of 21 consecutive days.
4. Vehicle + Aspirin Group: Pregnant rats were injected with the vehicle (saline) on E12.5, and the offspring received aspirin at a dose of 50 mg/kg through gavage for 21 consecutive days after weaning (Figure 1).

VPA and aspirin were dissolved in saline, and were administered at specified volume per weight.

### 2.2. Measurement of Growth and Maturation Postnatal

This measurement was based on weight gain from day 13 to 50 and included scoring the opening of the eyes from day 13 to 16 after birth. If both eyes opened, a score of 2 was given; if one eye opened, a score of 1 was given; and when neither eye opened, no score was awarded [23].

### 2.3. Behavioral Tests

**Figure 2:**
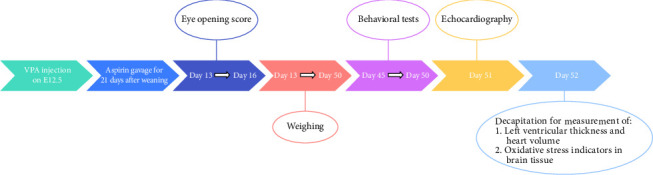
Schematic diagram of the experimental protocol.

Behavioral tests were conducted from all groups during puberty (day 45 to 50). Behavioral tests included a novel object recognition test (NORT) to measure cognition and a social interaction test to measure pseudo-autism activity (Figure 2). The number of rats used in behavioral tests was 7 per group, and 5 brain tissues in each group were used for the oxidative stress test.

#### 2.3.1. Social Behavioral Test

The social interaction test was conducted for 10 min in a rectangular area, which was divided into three sections by walls. Two wire cage-like containers, large enough for a rat to fit inside, were placed in the two side compartments. Initially, to familiarize the rat with the test environment, it was placed in the central area for 10 min to acclimate. A novel animal (of the same age) was placed in the wire cage on either the left or right side. This new rat represented the unfamiliar area 1, while the other wire cage remained empty (the empty area). The ratio of time spent in the area containing the unfamiliar rat 1 compared to the time spent in the empty area was measured and expressed as a sociability index (SI).

SI = (Time spent around unfamiliar rat/Time spent around empty area).

After the sociability test, a social preference test was conducted for another 10 min. In this phase, another new animal was introduced into the empty area under the wire cage, designated as unfamiliar area 2. The social preference index (SPI) was determined by using the formula that compared the duration spent close to the unfamiliar rat with the duration spent near the familiar rat [24].

SPI  = (Time spent around unfamiliar rat/Time spent around familiar rat).

#### 2.3.2. NORT

This test was conducted using the innate tendency of rodents to explore a novel object more than a familiar object with which they had previously been acquainted. We utilized two plastic playthings, nontoxic, odorless, around 5 cm tall and had a diameter of 4–6 cm comprising a tiny cube and a circular figurine featuring recognizable shapes.

A camera was positioned above the chamber along with a video camera to observe and document the animal's behavior.

To guarantee the accuracy of memory performance assessment in NORT, a minimum threshold for exploration time was implemented. Only animals that spent a minimum of 20 s exploring the object in total during the testing phase were considered for the analysis. In our research, every participant exceeded this limit, so no exclusions were needed.

This approach was carried out in three stages: habituation, training, and testing, within a tranquil setting with uniform lighting.

During the habituation phase, each rat was introduced into the vacant chamber devoid of any items and was permitted to explore the inner area freely for 10 min. During the training phase, which occurred 24 h post the initial phase, two identical cubes were positioned in the chamber 5 cm away from the walls, permitting the rat to investigate the objects for 10 min.

On the day of the test, 24 h after getting acquainted with the two identical items, one item was swapped out for a different object. The items were situated in comparable spots, and every rat was subsequently permitted to investigate the chamber for 10 min. The parameters assessed in this experiment included the recognition index and difference score, computed in the following way:

Recognition index: (Time devoted to examining the new object in the third phase divided by the overall time spent exploring both objects in the third phase) × 100.

Difference score: To assess this index, the time disparity between the new and old items was determined. The recognition of the new object was indicated by a greater duration spent near the new object relative to the old object, resulting in a typically positive difference score and a recognition index that surpassed 50% [25].

### 2.4. Echocardiography

Echocardiography was performed 24 h after behavioral tests at PND51. To conduct this test, the rat was first sedated using a tranquilizer (ketamine at a dosage of 10 mg/kg). After the rats were immobilized and their chest area was shaved, ultrasonography was performed starting from the chest cavity and extending beyond the space between the fifth rib using specialized gels. The images obtained were stored by the device, and the desired indices (left ventricular thickness, and heart rate) were measured and recorded using the device's software.

After the animal was placed on the table, echocardiography was performed using M-mode and pulse wave with a 10 MHz microconvex transducer while the animal was restrained in a supine position, slightly inclined to the right or left side. The surface of the chest and the transducer were coated with ultrasound gel. By moving the transducer over the chest, an appropriate image of the heart was obtained and recorded [26].

### 2.5. Measurement of Left Ventricular Thickness and Heart Volume

The thickness of the left ventricle was measured using a digital caliper, and the heart volume was measured using a syringe filled with water.

#### 2.5.1. Measurement of Left Ventricular Thickness

The thickness of the left ventricle was measured once with an echocardiography device and its corresponding software, and again after euthanizing the rats, it was measured from the thickest area using a digital caliper.

#### 2.5.2. Measurement of Heart Volume by Displacement of Fluid

The method for measuring heart volume using a syringe and the change in water volume in laboratory rats was used as an experimental technique to estimate heart volume in small animals like rats. To perform this method, a 5-cc syringe and water were required. First, the syringe was filled with a specific volume of water, and this volume was measured. Then, the heart was placed inside the syringe, and the volume was measured again. The difference between this volume and the volume of the syringe without the heart represented the heart volume [27].

### 2.6. Measurement of Oxidative Stress

Oxidative stress indicators included measuring total antioxidant capacity (TAC), total oxidant status (TOS), oxidative stress index (OSI), GSH, and GSSG in brain tissue.

#### 2.6.1. TAC

Samples (10 μL) (plasma/milk/urine/feed extract, etc.) were mixed with 250 mL of working FRAP reagent and absorbance (593 nm) was measured at 0 min after vortexing.

After that, samples were placed at 37 C in a water bath, and absorption was measured again after 4 min.

Results were calculated as follows:

FRAP value of sample (μM) = (Change in absorbance of sample from 0 to 4 min/Change in absorbance of standard from 0 to 4 min) × FRAP value of the standard [28].

Ascorbic acid was used as a well-characterized antioxidant and served as a reference standard in various antioxidant assays. By expressing TAC in terms of vitamin C equivalency, the study provided a familiar context for interpreting the antioxidant capacity of different samples.

#### 2.6.2. TOS

Oxidants present in the sample converted the ferrous ion–o-dianisidine complex into ferric ion. The oxidation reaction was facilitated by glycerol molecules, which were abundant in the reaction medium. The ferric ion formed a colored complex with xylenol orange in an acidic environment. The color intensity, measurable through spectrophotometry, correlated with the overall quantity of oxidant molecules in the sample. The assay was standardized using hydrogen peroxide, and the outcomes were reported as micromolar hydrogen peroxide equivalents per liter (μmol H_2_O_2_ Equiv./L). OSI was used to assess the balance between oxidative stress and antioxidant defense in biological systems. OSI was calculated using the following formula: OSI (arbitrary units) = [(TOS, μmolH_2_O_2_equiv./L)/(TAS, μmol Vit C equiv./L) × 100] [29].

#### 2.6.3. Measurement of Reduced Glutathione (GSH)

The principle of the assay was that the sulfhydryl group of glutathione reacted with 5, 5′-dithiobis (2-nitrobenzoic acid) (DTNB), producing a yellow derivative known as 5-thio-2-nitrobenzoic acid (TNB). The mixed disulfide, GSTNB (GSH and TNB), which was produced simultaneously, was reduced by glutathione reductase to yield more TNB. The quantity of TNB generated was directly related to the concentration of GSH present in the sample [30].

#### 2.6.4. Measurement of Oxidized Glutathione (GSSG)

The principle of measuring GSSG with this method involved reducing the GSSG present in the sample to GSH in the presence of reducing agents (NaBH4 and NaOH), measuring total GSH, and then calculating the amount of GSSG based on the difference between total GSH and initial GSH in the sample. The initial GSH content in the tissue was subtracted from the total GSH content (GSH + GSSG), and the final amount of GSSG was calculated [31].

### 2.7. Statistical Analysis

All data were shown as mean ± standard error of the mean (mean ± SEM). Statistical analysis and graphing were conducted using GraphPad Prism 9 software, with a significance threshold set at *p* < 0.05. The differences in various parameters among the experimental groups were analyzed using one-way ANOVA, followed by Tukey's post hoc test.

## 3. Results

The results were divided into five sections, which included the examination of the effect of aspirin following the induction of an autism model with VPA on: (1) cognition, (2) social behavior, (3) average weight and eye-opening scores, (4) left ventricular hypertrophy, and (5) oxidative stress in adult male rats.

### 3.1. Cognitive Results

NORT was utilized to assess cognition. In this assessment, two indices were used to evaluate memory: (1) recognition index and (2) time difference score.

#### 3.1.1. Outcomes of the Recognition Index

**Figure 3:**
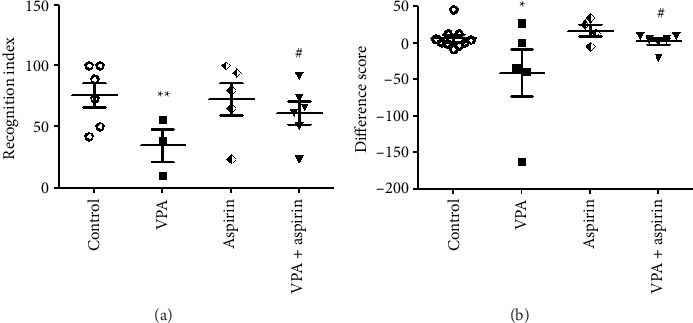
Comparison of the average percentage of cognitive index (a) and difference score (b) among the examined groups. ^∗^ and ^∗∗^ denote significant differences at the levels of *p* < 0.05 and *p* < 0.01 when compared with the control group. ^#^ signifies a difference between the VPA groups receiving aspirin versus the VPA group (*p* < 0.05). Data are shown as mean ± SEM (*n* = 7).

The data presented in Figure 3(a) revealed that, in the majority of rats from the VPA group, the proportion of time spent near the new object in relation to the total exploration time for both objects was under 50%, with one exception. Conversely, the majority of rats in the treatment groups devoted more than 50% of their time investigating the unfamiliar object. The recognition index for the VPA group demonstrated a noticeable reduction in comparison to the control group (*p* < 0.01). This index showed a significant rise in the VPA group receiving aspirin in comparison to the VPA group (*p* < 0.05) “*F* (16, 3) = 7.05, *p*=0.031” (Figure 3(a)).

#### 3.1.2. Results of the Time Difference Score

As illustrated in Figure 3(b), the average time difference between the new and old objects in the majority of rats from the VPA group, with one exception, was under zero and leaned toward negative values. Conversely, the majority of rats in the treatment groups showed data above zero and positive. According to the obtained results, the VPA group spent less time around the new object and more time near the old object compared to the control group (*p* < 0.05). Meanwhile, the time spent around the new object compared to the old object in the treated groups significantly increased compared to the VPA group (*p* < 0.05) “*F* (20, 3) = 10.76, *p*=0.016” (Figure 3(b)).

### 3.2. Social Behavior

To evaluate social behavior, two indices were measured: social interaction and SPI.

#### 3.2.1. Results of SI

**Figure 4:**
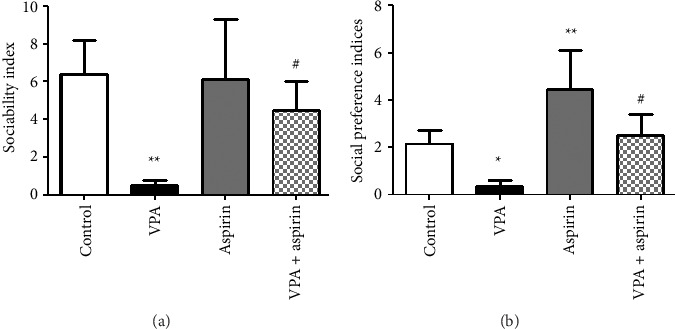
Comparison of the average sociability index (a) and social preference index (b). ^∗^ and ^∗∗^ denote significant differences at *p* < 0.05 and *p* < 0.01 between the VPA group and the control group. ^#^ signifies a difference between the VPA groups given aspirin compared to the VPA group (*p* < 0.05). Data are shown as mean ± SEM (*n* = 7).

The findings of this study indicated that the most time spent around the familiar rat was observed in the VPA group, with a notable difference between the VPA group and the control group. The social interaction index in the VPA group displayed a notable reduction in comparison to the control group (*p* < 0.01). No notable differences were seen between the other groups and the control group. In the meantime, the time spent near the unfamiliar rat and the social interaction index in the aspirin-treated VPA groups exhibited a notable rise when compared to the VPA group (*p* < 0.05) “*F* (15, 3) = 4.13, *p*=0.019” (Figure 4(a)).

#### 3.2.2. Results of SPI

SPI in the VPA group exhibited a notable decline compared to the control group (*p* < 0.05), suggesting that the proportion of time spent near the unfamiliar rat compared to the familiar rat was lesser in the VPA group than in the control group. In contrast, this index showed a significant increase in the aspirin group compared to the control group, with rat in the aspirin group spending more time around the stranger rat than those in the control group (*p* < 0.01). SPI in the aspirin-treated VPA group significantly increased compared to the VPA group, with rat in the aspirin-treated VPA group spending more time around the stranger mouse relative to the familiar one than those in the VPA group (*p* < 0.05). No significant differences were observed between other groups and the control group “*F* (19, 3) = 5.18, *p*=0.021” (Figure 4(b)).

### 3.3. Average Weight and Eye-Opening Scores

In this section, the results related to weight measurement from day 13 to 50 and the scoring of eye opening from day 13 to 16 after birth were presented.

#### 3.3.1. Results of Average Body Weight

**Figure 5:**
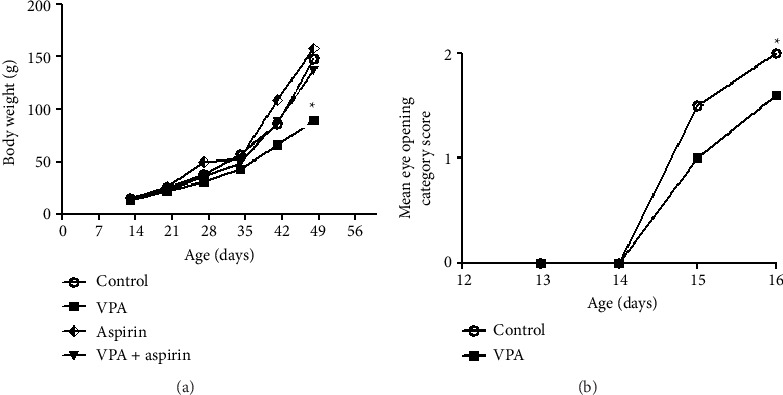
Comparison of the average body weight from Day 13 to 50 (a) and eye opening scores from Day 13 to 16 after birth (b). ^∗^ shows a notable difference at the level of (*p* < 0.05) between the VPA group and the control group (*p* < 0.05). Data are presented as mean ± SEM (*n* = 7).

According to the results of the experiment, the average weight of the rat showed an increasing trend from day 13 to 50 after birth in all groups. On day 50 after birth, a notable difference was noted between the VPA group and the control group. In particular, the VPA group's average weight was less than that of the control group (*p* < 0.05) “*F* (14, 3) = 2.57, *p*=0.041” (Figure 5(a)).

#### 3.3.2. Results of Eye Opening Scores

As shown in Figure 5(b), only on day 16 a notable difference was observed when comparing the VPA group to the control group, as the VPA group had a considerably lower score for eye-opening than the control group (*p* < 0.05).

### 3.4. Results of Left Ventricular Hypertrophy

To assess left ventricular hypertrophy, the thickness of the left ventricle was measured once using echocardiography and the relevant software, and again after euthanizing the rat, using a digital caliper. The heart volume and heart rate were also measured.

#### 3.4.1. Results of Left Ventricular Thickness Using Digital Caliper

**Figure 6:**
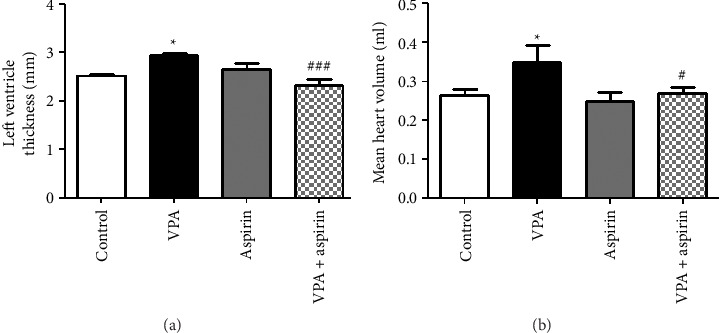
Comparison of mean left ventricular thickness using digital caliper (a) and mean heart volume (b). ^∗^ indicates a significant difference at the level of (*p* < 0.05) between the VPA group and the control group, and ^#^ indicates a difference between the VPA + aspirin group and the VPA group at the level of (*p* < 0.05). Data are presented as mean ± SEM (*n* = 5).

As illustrated in Figure 6(a), the left ventricle thickness in the VPA group was considerably greater than that of the control group (*p* < 0.05), and in the aspirin-treated VPA group, a significant decrease was observed compared to the VPA group “*F* (18, 3) = 8.28, *p*=0.027” (*p* < 0.05).

#### 3.4.2. Results of Heart Volume

The heart volume in the VPA group was also significantly higher than the control group (*p* < 0.05), and in the aspirin-treated VPA group, a significant decrease was found compared to the VPA group “*F* (25, 3) = 5.23, *p*=0.031” (Figure 6(b)).

#### 3.4.3. Results of Left Ventricular Thickness Using Echocardiography

**Figure 7:**
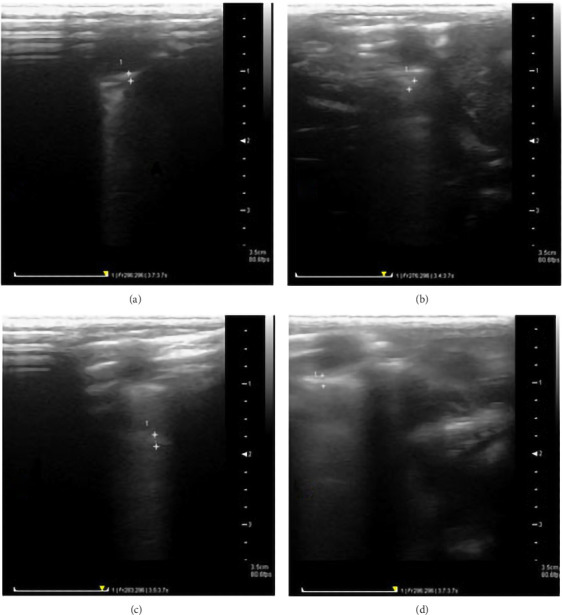
Comparison of changes in left ventricular thickness in the studied groups. (a) Control group, (b) aspirin group, (c) VPA group, and (d) treated or aspirin group.

**Figure 8:**
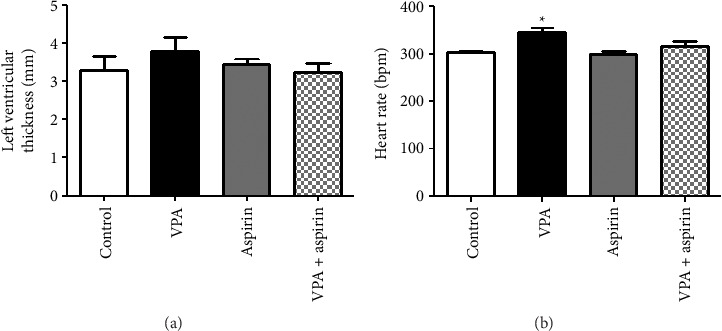
Comparison of average left ventricular thickness and heart rate. ^∗^ denotes a notable difference at the level of (*p* < 0.05) between the VPA group and the control group. The variations among the data are shown as mean ± SEM (*n* = 7).

The results of the mean left ventricular thickness using echocardiography indicated that the left ventricular thickness in the VPA group (Figure 7(c)) had increased compared to control (Figure 7(a)), aspirin (Figure 7(b)) and VPA + aspirin (Figure 7(d)) groups; however, this increase was not statistically significant “*F* (28, 3) = 2.64, *p* = 0.56” (Figures 7 and 8(a)).

#### 3.4.4. Results of Heart Rate Using Echocardiography

**Figure 9:**
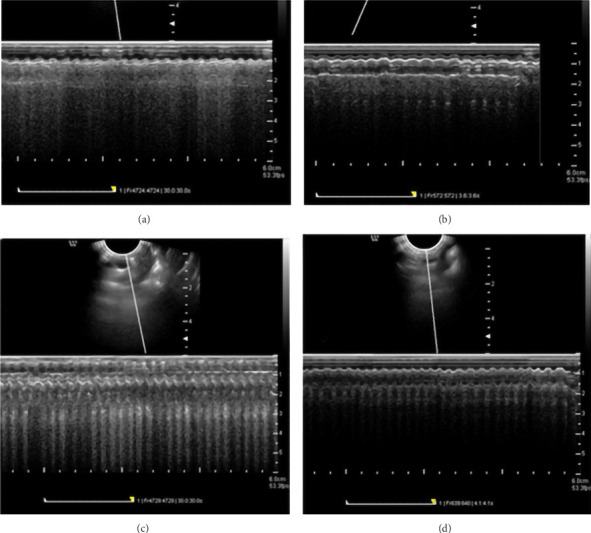
Comparison of heart rate changes in the studied groups. (a) Control group, (b) aspirin group, (c) VPA group, and (d) VPA group treated with aspirin.

The heart rate in the VPA group (Figure 9(c)) showed a significant increase compared to the control group (Figure 9(a)), while no significant differences were observed between VPA group, aspirin (Figure 9(b)) and VPA group treated with aspirin (Figure 9(d)). “*F* (33, 3) = 4.28, *p*=0.0002” (Figures 8(b) and 9).

### 3.5. Results of Oxidative Stress

To evaluate oxidative stress, the levels of reduced glutathione (GSH) and oxidized glutathione (GSSG), TAC, TOS, and OSI were measured.

#### 3.5.1. Results of GSH

**Figure 10:**
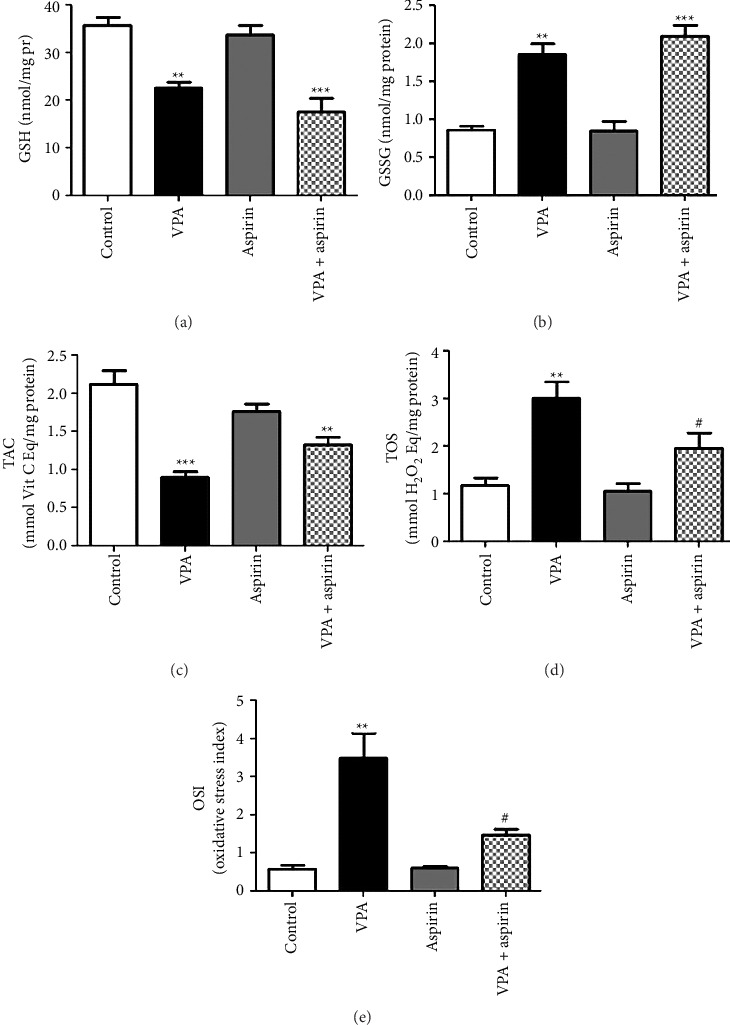
The comparison of average GSH (a), GSSG (b), TAC (c), TOS (d), and OSI (e) among the experimental groups. ^∗∗^ and ^∗∗∗^ signify significant differences at (*p* < 0.01) and (*p* < 0.001) between the experimental groups relative to the control group. ^##^ denotes a significant difference at (*p* < 0.01) between the VPA group treated with aspirin and the VPA group (*n* = 5).

The mean level of reduced glutathione in the VPA group showed a significant decrease in comparison to the control group (*p* < 0.01), and a notable decrease was also recorded in the VPA group receiving aspirin when compared with the control group (*p* < 0.001) “*F* (8, 3) = 31.16, *p*=0.0001” (Figure 10(a)).

#### 3.5.2. Results of GSSG

The mean level of oxidized glutathione in the VPA group showed a significant increase compared to the control group (*p* < 0.01), and a significant increase was also found in the VPA group treated with aspirin compared to the control group (*p* < 0.01) “*F* (8, 3) = 31.16, *p*=0.0001” (Figure 10(b)).

#### 3.5.3. TAC Results

Figure 10(c) indicates that the overall antioxidant level in the VPA group was reduced compared to other groups, and this disparity relative to the control group was significant (*p* < 0.001). The overall antioxidant factor also exhibited a notable reduction in the VPA + aspirin group in comparison to the control group “*F* (8, 3) = 21.37, *p*=0.0004” (*p* < 0.01).

#### 3.5.4. Results of TOS

The total oxidant factor in the VPA group showed a significant increase compared to the control group (*p* < 0.01). Meanwhile, the total oxidant factor in the VPA + aspirin group showed a significant decrease compared to the VPA group (*p* < 0.05) “*F* (8, 3) = 11.88, *p*=0.0026” (Figure 10(d)).

#### 3.5.5. OSI Results

OSI, derived from the ratio of TOS/TAC, demonstrated a notable rise in the VPA group in comparison to the control group (*p* < 0.01), and a significant reduction was noted in the VPA group that received aspirin relative to the VPA group (*p* < 0.05) “*F* (8, 3) = 16.91, *p*=0.0008” (Figure 10(e)).

## 4. Discussion

The current research showed that administering low-dose aspirin enhanced social behavior and cognitive functioning in rats that were prenatally exposed to VPA, a recognized model of ASD. These results indicate a possible neuroprotective and regulatory function of aspirin in mitigating ASD-related impairments, influenced by its impact on oxidative stress, neuroinflammation, and cardiac performance.

Rats treated with aspirin showed increased social interaction and fewer stereotypic behaviors than untreated rats exposed to VPA. This enhancement corresponds with the drug's established anti-inflammatory and antioxidant characteristics, implying that the alteration of neuroinflammatory processes might explain the seen behavioral improvements. Our data specifically showed lower levels of oxidative stress markers in the group treated with aspirin, suggesting that redox regulation plays a role in improving ASD-like symptoms.

At the cellular level, the reduction of cerebellar Purkinje cells in ASD models has been linked to impairments in emotional and social processing [32].

Research indicates that the activity of the GABAergic system in the brains of individuals with autism is diminished, potentially leading to fewer social interactions, heightened repetitive behaviors, and modified sensory-motor functions in children with autism [33].

Since aspirin seemed to reduce behavioral impairments, it could provide indirect advantages by maintaining cerebellar integrity or influencing downstream signaling. The noted alterations in the GSH/GSSG ratio further reinforce the idea that aspirin can restore oxidative homeostasis, a pathway frequently disrupted in ASD [34].

Reduced GSH levels and indicators of higher oxidative stress associate with the severity of ASD [14]. Furthermore, glutamate excitotoxicity can lead to neuroinflammation through microglial activation and the release of inflammatory mediators [35]. Excessive production of ROS may disrupt DNA methylation and cause a positive feedback mechanism [36]. Therefore, patients with ASD are more vulnerable to oxidative stress and neurotoxicity.

Enhancements in working memory and attentional control after aspirin treatment highlight its wider impacts on corticocortical and corticolimbic networks [37]. Dysfunctional activity in the medial prefrontal cortex and hippocampus—essential areas for executive function and emotional control—is well established in ASD, and our results indicate that aspirin might partially rejuvenate activity within these networks [38].

This research also revealed changes in left ventricular thickness, heart rate, and volume in rats exposed to VPA, which were corrected with aspirin treatment. These findings indicate an additional advantage of low-dose aspirin in managing autonomic dysfunction associated with ASD. Aspirin may lessen cardiovascular risks frequently neglected in neurodevelopmental models by decreasing platelet aggregation and oxidative stress [39]. Researchers have studied various indicators of cardiac function in autism, including heart rate variability, changes in heart rate, and the coordination between heart rate and respiration [6]. Studies have indicated that the thickness of the heart muscle, especially in the left ventricle, varies in ASD, and these variations can influence the heart's pumping efficiency [40]. Patients with ASD also exhibit differences in ejection fraction, diastolic function, and systolic function [41]. The current findings indicated that low-dose aspirin decreased left ventricular thickness, heart volume, and heart rate in the VPA group.

While ASA does not directly influence cardiovascular function, it has been noted to provide effective protection against pathological cardiovascular conditions such as atherosclerosis, ischemic heart disease, and myocardial infarction [42]. One of the protective mechanisms of aspirin on cardiovascular function is the inhibition of platelet activity by aspirin [43]. By reducing platelet aggregation, it helps prevent blood clot formation in arteries and the occurrence of diseases such as heart attacks and strokes [43]. Aspirin is often used as a preventive measure to reduce the likelihood of blood clot formation in arteries, which can improve overall cardiac function [44].

While aspirin has antioxidant and anti-inflammatory effects, this study mainly examined oxidative stress indicators and did not evaluate inflammatory markers like TNF-α, IL-6, or CRP. Thus, although the observed enhancements in behavior and heart function likely engage both antioxidant and anti-inflammatory mechanisms, we cannot conclusively ascertain the specific contribution of each pathway. This signifies a constraint of the present research and emphasizes the necessity for upcoming studies that integrate both oxidative and inflammatory markers to elucidate aspirin's varied therapeutic function in neurodevelopmental and cardiovascular areas.

Taken together, our results offer strong evidence for the diverse impacts of aspirin in an ASD model, covering behavioral, molecular, and systemic areas. Although earlier research has produced inconsistent findings on aspirin's effect on cognition, our data endorse its possible role in regulating oxidative stress and redox balance, which may have consequences for ASD treatment. Nevertheless, dosage, duration, and timing of development need to be meticulously evaluated to prevent confounding effects. Subsequent studies need to investigate if aspirin's advantages apply to other ASD models and whether combined effects occur when used with agents aimed at glutamate excitotoxicity, microglial activation, or mitochondrial function.

## 5. Conclusions

According to the results of this study, VPA injection during pregnancy led to an impairment in social behavior, and cognition, increased oxidative stress, and cardiac function problems in adult offspring. However, low doses of aspirin for 21 days significantly improved cognition, social behavior, and cardiac function in VPA-affected rats by reducing oxidative stress.

## Data Availability

The datasets used and/or analyzed during the current study will be available from the corresponding author upon reasonable request.
